# Accurate Detection of Recombinant Breakpoints in Whole-Genome Alignments

**DOI:** 10.1371/journal.pcbi.1000318

**Published:** 2009-03-20

**Authors:** Oscar Westesson, Ian Holmes

**Affiliations:** Department of Bioengineering, University of California Berkeley, Berkeley, California, United States of America; Broad Institute of MIT and Harvard, United States of America

## Abstract

We propose a novel method for detecting sites of molecular recombination in multiple alignments. Our approach is a compromise between previous extremes of computationally prohibitive but mathematically rigorous methods and imprecise heuristic methods. Using a combined algorithm for estimating tree structure and hidden Markov model parameters, our program detects changes in phylogenetic tree topology over a multiple sequence alignment. We evaluate our method on benchmark datasets from previous studies on two recombinant pathogens, *Neisseria* and HIV-1, as well as simulated data. We show that we are not only able to detect recombinant regions of vastly different sizes but also the location of breakpoints with great accuracy. We show that our method does well inferring recombination breakpoints while at the same time maintaining practicality for larger datasets. In all cases, we confirm the breakpoint predictions of previous studies, and in many cases we offer novel predictions.

## Author Summary

In viral and bacterial pathogens, recombination has the ability to combine fitness-enhancing mutations. Accurate characterization of recombinant breakpoints in newly sequenced strains can provide information about the role of this process in evolution, for example, in immune evasion. Of particular interest are situations of an admixture of pathogen subspecies, recombination between whose genomes may change the apparent phylogenetic tree topology in different regions of a multiple-genome alignment. We describe an algorithm that can pinpoint recombination breakpoints to greater accuracy than previous methods, allowing detection of both short recombinant regions and long-range multiple crossovers. The algorithm is appropriate for the analysis of fast-evolving pathogen sequences where repeated substitutions may be observed at a single site in a multiple alignment (violating the “infinite sites” assumption inherent to some other breakpoint-detection algorithms). Simulations demonstrate the practicality of our implementation for alignments of longer sequences and more taxa than previous methods.

## Introduction

Recombination is the process by which a child inherits a mosaic of genes or sequences from multiple parents. Though most species participate in some form of genetic mixing or recombination, the mechanics by which this occurs varies greatly among them. In higher order organisms, crossing over occurs in meiosis along the parent-child relationship, whereas in bacteria, viruses, and protozoans, homologous exchange of DNA material can occur from one individual to another without the need for sexual reproduction [1]. The diversity with which recombination occurs motivates the need for different models and methods, each ideally suited to its biological situaion. We have developed a probabilistic approach to recombination detection that we believe to be superior for analyzing situations of admixture of pathogen subspecies with a high mutation/recombination ratio.

The situation we concern ourselves with has been termed *phylogenetic recombination inference* (PRI) by [2], and works by inferring phylogenetic tree topology changes over a multiple alignment. Though it has been shown that under a neutral coalescent model, the number of recombination events which will lead to a tree topology change is very small, [3] in situations of admixture following geographical separation a greater proportion of topology-changing recombinations are expected. Abandoning the infinite-sites model of sequence evolution and instead using a continuous-time Markov chain makes direct inference intractable, and so we instead employ a phylo-HMM which models an *effect* of recombination, rather than modeling the process explicitly.

While recombination detection is an interesting mathematical challenge, fast, flexible, and reliable computational methods are also motivated by a multitude of biological reasons. We see our method not as being able to answer all of these biological questions on recombination, but rather a potentially valuable tool for furthering recombination research.

- **Genome Dynamics** The two most significant factors driving change in genomes in the context of evolutionary adaptation and diversity are point mutation and recombination. The ratio between these two differs greatly among organisms; in most, recombination among subtypes is fairly rare and point mutation occurs comparatively often. Similarly to point mutation, recombination has the possibility to combine independent fitness-enhancing changes among genomes as well as disable genes. As Awadalla remarks, “recombinant genomes are known to be associated with changes in phenotype or fitness, including heightened or reduced pathogenicity or virulence” [1]. Our understanding about where, how, and why recombination occurs is comparatively primitive. We know, for example, that pathogens such as *Chlamydia trachomatis* have recombination hotspots [4], but the relevant *cis*-acting factors are unknown. The precise determination of breakpoints in recombining pathogens is crucial for higher-level downstream analyses such as that of [4], or the methods proposed by [2] and [5] in which genome-scale conclusions about recombination are made from large sets of observed breakpoint locations. We believe our method offers improved precision and flexibility as compared to other programs. Furthermore, in light of the high proportion of HIV isolates which are recombinant, it can be useful that PRI allows one to safely relax the requirement that all but one of the sequences in the alignment are ‘pure’ subtypes.
  As well as being an appealing scientific challenge, a better understanding of the dynamics of pathogen genome evolution might help highlight molecular processes to target in designing therapeutics, as well as opening up the possibility for genetic manipulation.
- **Phylogenetic Analysis** When performing phylogenetic analysis on a multiple sequence alignment, most methods assume that there is a unique hierarchical relationship among the taxa in question. If recombination has occurred in evolutionary history, this phylogeny reconstruction will be systematically faulty in either its topology, branch lengths or both. Incorrect trees could hinder further comparative genomic inferences made from the data [6]. In our training scheme, we estimate separate trees for all regions in the alignment, and if more sophisticated tree-inference methods ought to be used, our precise breakpoint inference allows for training trees on the alignment sections.
- **Genetic Mapping** In using genetic mapping information to locate genes associated with various phenotypes, it is vital to know the extent of genome rearrangement present. For a discussion of how this can affect microbial pathogen analysis, see [7].

### Previous Related Work

We give here an outline of previous methods which are related to our phylo-HMM approach. For a more thorough survey or recombination detection methods, see [1].

The rationale for phylogenetic recombination inference is motivated by the structure of the Ancestral Recombination Graph (ARG), which contains all phylogenetic and recombination histories. The underlying idea is that recombination events in the history of the ARG will, in certain cases, lead to discordant phylogenetic histories for present-day species.

Various approaches to learn the ARG directly from sequence data have been developed, such as [8] and [9]. We recognize that PRI is in a somewhat different category both in goal and approach as compared these methods, though they are motivated by the same underlying biological phenomenon. Rather than aiming to reconstruct the ARG in its entirety, our emphasis is on modeling fast-evolving organisms with the goal of accurately detecting breakpoints for biological and epidemiological study.

The most widely-used program for phylogenetic recombination detection is SimPlot [10] (on MS Windows). Recombination Identification Program (RIP), a similar program, [11] runs on UNIX machines as well as from a server on the LANL HIV Database site. This program slides a window along the alignment and plots the similarity of a query sequence to a panel of reference sequences. The window and step size are adjustable to accommodate varying levels of sensitivity. Bootscanning slides a window and performs many replicates of bootstrapped phylogenetic trees in each window, and plots the percentage of trees which show clustering between the query sequence and the given reference sequence. Bootscanning produces similar output to our program, namely a predicted partition of the alignment as well as trees for each region, but the method is entirely different.

In [12], Husmeier and Wright use a model that is similar to ours except for the training scheme. Since they have no scalable tree-optimizing heuristic, their input alignment is limited to 4 taxa so as to cover all unrooted tree topologies with only 3 HMM states, making their method intractable for larger datasets. They show they are able to convincingly detect small recombinant regions in *Neisseria* as well as simulated datasets limited to 4 taxa [12].

The recombination detection problem can be thought of as two inter-related problems: how to accurately partition the alignment and how to construct trees on each region. This property is due to the dual nature of the ARG: it simultaneously encodes the marginal tree topologies as well as where they occur in the alignment. Notice that if the solution to one sub-problem is known, the other becomes easy. If an alignment is already partitioned, simply run a tree-inference program on the separate regions and this will give the marginal trees of the sample. If the trees are known, simply construct an HMM with one tree in each state and run the forward/backward algorithm to infer breakpoints. Previous methods have used this property by assuming one of these problems to be solved and focusing on the other. For example, in Husmeier and Wright's model, there were very few trees to be tested, and so the main difficulty was partitioning the alignment, which they did with a HMM similar to ours. In SimPlot, windows (which are essentially small partitions) passed along the alignment and trees/similarity plots are constructed. This allows the program to focus on tree-construction (usually done with bootstrapped neighbor-joining) rather than searching for the optimal alignment partition.

By employing a robust probabilistic model with a novel training scheme, we find a middle ground between the heuristic approach of SimPlot [10] and the computational intractability of Husmeier and Wright's method [12], where we are essentially able to solve the recombination inference problem a whole, rather than neglecting one sub-part and focusing on the other. We use a HMM to model tree topology changes over the columns of a multiple alignment. This is done much in the same way as Husmeier and Wright, but our use of a more sophisticated tree-optimization (the structural EM heuristic) method allows searching for recombination from a larger pool of sequences. By modifying the usual EM method for estimating HMM parameters in a suitable way, we are able to simultaneously learn the optimal partitioning of the alignment as well as trees in each of these partitions. We are able to detect short recombinant regions better than previous methods for several reasons. First, we do not use any sliding windows which may be too coarse-grained to detect such small regions of differing topology. Second, our method allows each tree after EM convergence to be evaluated at every column, and so small recombinant regions are not limited by their size; they must only ‘match’ the topology to be detected or contribute to the tree training. By embedding trees in hidden states of an HMM, the transition matrix allows us to essentially put a prior on the number of breakpoints, as opposed to considering each column independently. Furthermore, since the counts in the E-Step are computed using all columns of the alignment, distant regions of the alignment with similar topology may contribute their signal to a single tree, whereas in a window-sliding approach each window is analyzed independently.

## Results

### Interpretation of the Results

**Figure 1:**
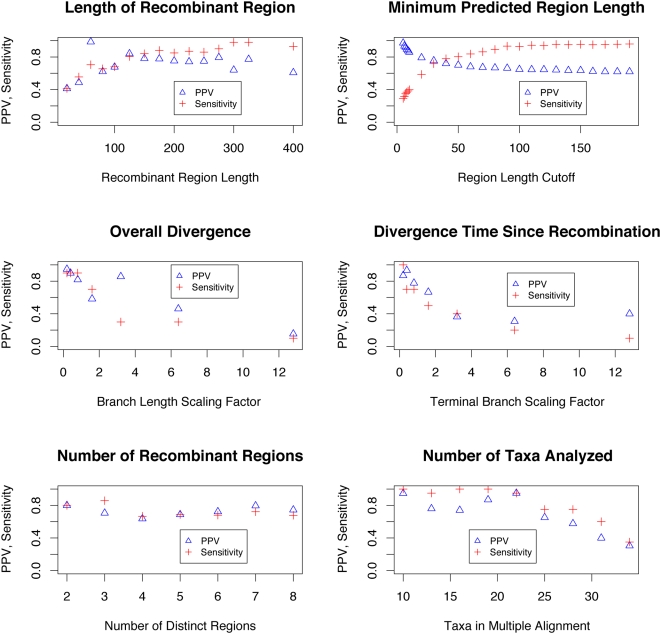
Accuracy of breakpoint detection varies as a function of simulation and inference parameters. In each case, we plot both positive predictive value (TP/(TP+FP) = PPV ) and sensitivity (TP/(TP+FN)). A correctly predicted breakpoint is defined as one which occurs less than 10 bp from a true breakpoint. We observe that the overall accuracy remains high except for situations of high diversity, extremely short recombinant region (less than 50 bp), or more than 20 taxa. In several cases, we were resource-limited and only able to provide a few data points for each variable, and this is the reason for the sparseness of the plots. Each data point is the maximum-likelihood outcome of 10 independently run EM trials, each one taking on average 15 minutes for small length/taxa, though this varies as seen in Figure 9.

Since it is difficult to experimentally verify predictions of recombination, we test our methods on previously-analyzed data from earlier studies on *Neisseria* and HIV-1 as well as simulated datasets. Individual cases highlighting various facets of our method can be seen in Text S1, whereas statistics summarizing simulations with respect to several simulation parameters are included in Figure 1.

When comparing real and simulated data, one must keep in mind that real data may have complications such as rate heterogeneity and structural features that are not present in simulations, which are carried out using a simple independent-sites Markov chain model of nucleotide evolution, such as the HKY85 model [13]. While this is currently the only model we use in our program, it is straightforward to extend this to other models.

In analyzing real data, when there were several alignments in the original analysis, we include only those in which we recover new breakpoints, or otherwise demonstrate our method's utility. It is implicitly assumed that in the analyses which we don't include, we came to similar or identical conclusions as the original authors.

In our analysis of simulated data, we aim to quantitatively characterize the strengths and weaknesses of the recHMM method by varying several simulation and analysis parameters. In each simulation case, ARGs (and hence 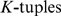 of trees) were generated with RECODON [14], which uses a coalescent-based simulation approach (for exact simulation parameters, see Text S1). In keeping with the above discussion of PRI vs coalescent modeling, we filter out ARGs whose marginal trees are identical in topology using the **treecomp** program [15]. Thus, in all of the simulations, a perfect detector of topology change would find every breakpoint. After tree-simulation, we simulate alignments using Seq-Gen, which generates multiple alignments according to simple independent sites Markov chain models. The reason for this decoupling of tree and sequence simulation is that Seq-Gen allows for easier manipulation of the variables we're testing, namely length of region, divergence since recombination, and overall divergence (by way of branch-scaling.) [16].

After running our program on the simulated data to estimate parameters, recombinant regions are determined by a posterior decoding algorithm which we describe briefly in the Methods section and is fully outlined in Text S1. (We use posterior decoding as opposed to the Viterbi algorithm since we are primarly concerned with maximizing the expected number of correct column labelings as opposed to maximizing the probability that our state path is exactly correct.) As the notion of a ‘true negative’, a column which was correctly annotated as a non-breakpoint, is not meaningful in this case, we instead examine positive predictive value: 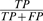, where a true positive (TP) is defined as a predicted breakpoint which occurs within 10 positions of a true breakpoint. Similarly, a false positive (FP) is a predicted breakpoint which has no true breakpoint within 10 positions. In plotting sensitivity: 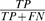, we define a false negative (FN) to be a true breakpoint for which we have no predicted breakpoint within 10 positions.

**Figure 2:**
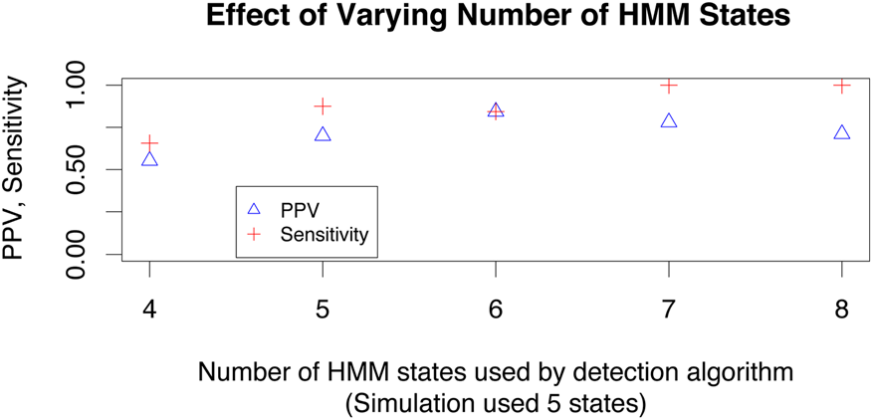
The detection power increases as more trees are added to the model. Here we analyze alignments with 5 regions, while setting our predicted number of states to various values. The sensitivity increases steadily while PPV tapers off at a fixed value.

We vary the following parameters with regard to simulation of data, the results of which are depicted in Figure 1:

- **Length of recombinant region** The length of the region which has a discordant phylogenetic signal is inversely proportional to detection power. We simulated alignments with three regions: two regions of length 200 bp on either side of the variable-length region, resulting in two true breakpoints, at positions 200 and 200+length(region).
  On alignments with recombinant regions longer than 100 bp, recHMM detects a high percentage of breakpoints with few false positives. Below 100 bp the detection suffered, with the program able to detect approximately 40% of breakpoints. For any recombination-detection program, smaller regions will be harder to detect, and so high accuracy down to 100 bp is promising.
- **Taxa** With more taxa, tree estimation becomes more difficult, and so distinguishing regions having different trees becomes more challenging. Further, comparing likelihoods of two large trees becomes unreliable as the scale of the likelihood becomes larger.
  In alignments with up to 23 taxa, detection is fairly strong, but begins to taper off around 25 taxa. Still, this is a notable improvement over Husmeier and Wright's model which could only accommodate 4 species. This is practically relevant only for initial screening for recombination; once the donor species are known, the alignment can be pruned of the irrelevant taxa for more accurate breakpoint detection.
- **Divergence** We vary the overall evolutionary time since speciation among the taxa by scaling the branch lengths of the tree used to simulate the alignment. The idea is that as divergence grows and the tree becomes indistinguishable from a ‘star-like’ topology, the phylogenetic signal relating species becomes weaker. On the other hand, if divergence were 0, the population would appear clonal (e.g. identity along alignment columns) and recombination would be undetectable.
- **Divergence since recombination event** In varying the divergence time since recombination, we wish to quantify the idea that more recent recombination events are easier to detect, since they have closer homology to their donor genomes.
  Since directly varying the divergence since the recombination event is difficult, we instead restrict our analysis to a subset of topology-changing ARGs, namely those whose marginal trees differ by a leaf-transfer event (as opposed to a general subtree-transfer). While this may be an unlikely scenario from a pure coalescent perspective, newly emergent recombinant pathogens can be represented as leaf-transfer events. In terms of simulation, this restriction of ARGs allows us to approximate scaling all branches (and sub-branches) since recombination by scaling only terminal branches, allowing us to demonstrate the difference in detection power between ‘ancient’ and ‘recent’ recombination events.
  Divergence and divergence since recombination appear to affect detection power in a similar way. Though it is difficult to draw conclusions from so few data points, one can see a sharper dependence in the leaf-scaling case, whereas in scaling all branches, the curve is slightly gentler. This can be intuitively understood considering that the leaf-scaling is varying only the relevant part of the tree, whereas when the whole tree is scaled, the effect on the phylogeny is more evenly dispersed, resulting in a more gradual effect on detection power.
- **Number of recombinant regions** 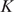 This varies the number of topologically distinct regions in the alignment. In analyzing these alignments, the number of HMM states, 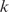, is set to the correct value 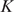.
  We observe that the method is relatively stable with respect to number of regions for the values we tested (2–8), provided that the number of regions is correctly specified. When this part of the model is mis-specified, the results are mixed, and we show results from simulation studies varying the model structure for fixed alignments in Figure 2. The 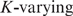 plot in Figure 1 does not take into account the possibility that non-bordering regions were wrongly annotated as coming from the same state. The breakpoints of these regions would still be detected, but their tree topology would be incorrect. Thus, we re-emphasize that recHMM is primarily a breakpoint-detection tool, and that if serious inferences are to be made from the trees within each hidden state, then more sophisticated tree construction methods should be used on the separate alignment regions.

We examined the effect of the following parameters in data analysis:

- **Predicted number of recombinant regions** For certain values of 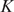 above, we vary 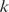 to see how detection power is affected when we have greater or fewer HMM states than distinct regions. We would have liked to vary 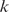 extensively for every value of 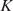, but we as were limited by computing time, we analyzed only 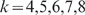 for 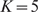.
  From this study, we concluded that specifying more states increases sensitivity, but at a slight cost of PPV. Intuitively, if a model has too few states, discordant regions may be merged together and modeled by a consensus topology, instead of being correctly modeled as separate recombinant regions with their own tree-states. If excessively many states are used, then presumably more of the genuinely differing regions will be modeled, but also small, spurious regions of convergent mutations or rate heterogeneity could be modeled by one of the extra states, leading to falsely predicted breakpoints. In many cases, the cutoff criterion helps in filtering out small errant regions, and we see only a moderate drop in PPV in Figure 2 for 7 and 8 states.
- **Length of cutoff criterion** The cutoff criterion is the value of the smallest distance between breakpoints we allow in our predicted state path. For a detailed description on how this cutoff criterion informs our posterior decoding algorithm, see the Methods section and Text S1. Simply stated, we disregard paths with breakpoints occurring within the cutoff of each other when choosing a maximal path.
  In Figure 1, we see that sensitivity rises as we allow for shorter region predictions (by specifying a higher cutoff value). PPV shows the opposite trend; with smaller cutoff criteria, we can be increasingly certain that any breakpoints we find are true breakpoints. The cutoff value where the two curves intersect can be thought of as a value which optimally balances sensitivity and PPV, and so in our simulated data analysis we set the cutoff to 30 bp.

In our analysis of real data, we cover a range of data sizes and types, ranging between 4 and 9 taxa, with length ranging from 700 bp to circa 10,000 bp. We find that in each case, we are able to recover the previous authors' predictions for breakpoints. In many cases, we find compelling evidence for additional, often shorter recombinant regions that the original analysis either missed completely or registered as minor ‘spikes’ in their plot. In each example we highlight the aspects of our method that contribute to its sensitivity, flexibility and utility. In the case where we had no additional predictions to add to a dataset, we omitted that analysis for brevity. For example, we analyzed the data from [4], but the low mutation rate enabled their simpler approach to adequately determine breakpoints. In this situation we acknowledge that our method is able, but not necessary, to analyze the data.

### *Neisseria* ArgF and penA Genes

We used our program to analyze data from *Neisseria* data that consisted of single gene regions suspected of recombination. In these analyses, recombinant regions were quite short and we demonstrate that our method is capable of handling this situation.

**Figure 3:**
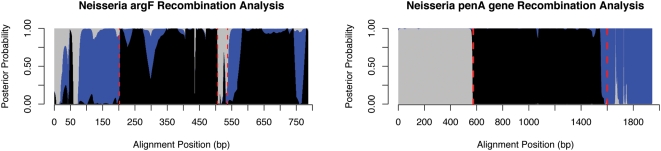
Analyses of Neisseria *argF* (left) and *penA* (right) data. The left plot shows the analysis of *Neisseria* argF data with predictions from Husmeier and Wright, who used a similar method, [12] in red dashed lines. We confirm each of their breakpoints and are able to better characterize uncertain regions. Still, the region from 0–75 remains difficult to characterize. Different colors represent posterior probabilities of different tree-topology states in the HMM, and sharp changes in color indicates likely recombination breakpoints. The right figure shows analysis of *Neisseria* penA data, an alignment of 9 taxa of length circa 1900 bp, demonstrating our ability to analyze many taxa. We confirm with high posterior probabilities the two breakpoints previously found by Bowler et al., shown in red [17].

In their 2001 work, Husmeier and Wright use a similar tree-topology HMM to detect recombination. Since each EM iteration involves searching over all possible tree topologies for the optimal trees for each region, they were limited to alignments of 4 taxa, where there are only 3 unrooted phylogenies [12]. As mentioned earlier, both this and window-based methods assume one part of the recombination inference problem to be solved. In this case, the method allocates one tree per HMM state, and so estimation of the trees is no longer necessary, leaving only the alignment partitioning problem to be solved. Our results on this dataset are shown in Figure 3. The previous predictions are shown in red dashed lines. The horizontal axis refers to the position within the alignment, and the vertical axis is partitioned according to posterior state probability of the HMM. The posterior state probability can intuitively be thought of as the probability that a certain column was generated by a certain phylogenetic tree, taking into account the model structure and all the alignment data. At each position, the posterior probabilities for the three trees must sum to one, and hence the different colors partition the vertical axis. We were able to closely replicate their results (namely the state probabilities depicted in Figure 15 of [12]).

In comparing our results to theirs, we note that our program, which does a probabilistic tree-updating step, rather than providing a hidden state for all possible topologies, recovers all the breakpoints of the previous study. At positions 202, 507, and 538 there are clearly points at which different colors have high posterior probabilites. In regions such as 0–50, it is difficult to make reliable inferences because with so few bases, phylogenetic tree construction is unreliable. As mentioned in the Methods section, we employ a simple length cutoff heuristic whereby all recombinant regions smaller than a certain length are removed. Though this is less sophisticated than, say, explicitly specifying a prior distribution over state paths which takes length into account, it performs reasonably well for the situations we analyzed. In considering putative crossovers, points where a tree with high posterior probability changes abruptly in favor of a different tree should be considered most closely. Also, topology changes that are extremely short could be the result of spurious convergent mutations, in which two phylogenetically distant species undergo mutations to the same base, making it seem as if they had exchanged information. Note also that our method is better able to characterize the regions 537–560 and 750–780. In [12], 537–560 is classified as “irregular”, and 750–780 shows only a spike in probability in Figure 13 of [12], and not at all in their Figure 15. We predict the 500–600 region to be composed of two separate topologies, and 750–780 to be a possibly newly characterized recombinant region, having the same topology as the 100–202 region.

In [17], Bowler et al. discovered a mosaic structure in the PenA gene of *Neisseria Meningitidis* which conferred resistance to Penicillin. Analyzing a DNA multiple alignment between 9 species, they were able to manually determine estimates for recombination breakpoints. Constructing phylogenetic trees for each of the regions gave them clues as to the donors of the acquired regions, after which these predictions were experimentally verified. In contrast, our method is able to simultaneously partition and estimate the trees of a recombinant alignment. In Figure 3 we see that our method predicts nearly the same breakpoints with high posterior probabilities. The alignment analyzed was composed of 9 species, covering the range of virulent and commensal *Neisseria* subtypes, with length 1950 bp. This analysis demonstrates the ability of our phylo-HMM to effectively make use of alignments with relatively many taxa, a notable advantage over Husmeier et al.'s method. For a quantitative look at how detection power varies with taxa number, see Figure 1. By using so many subtypes for comparison, Bowler et al. were able to precisely determine which species were the donors and recipients of the recombinant regions, and subsequently verified these predictions in a laboratory setting. If they had been limited to 4 taxa, the analysis would have had to be repeated many times to cover all the possibilities. Biologically, the results in [17] motivate a search for recombination *within* genes implicated in resistance, in contrast to the multiple resistance gene transfer that has been previously studied, and this is a possible application of our method.

### HIV-1 Whole-Genome Scans

In order to determine the effectiveness of our method on longer alignments, we analyzed several datasets of entire genomes (10,000 bp) of HIV-1 strains suspected of inter-subtype recombination. Our method is equally able to perform on the genome scale as it is on the single-gene scale. In *Neisseria* argF, one of the predicted recombinant regions was only 30 bp long, whereas in HIV they range from 100 bp to 6 kb. This is a notable advantage over sliding-window methods which have a fixed resolution to be used over the whole scan. We demonstrate here that we are able to determine breakpoints between both large and small recombinant regions, making our method a promising tool for comparative analysis of HIV and similar genomes. In analyzing data from previous studies, we recovered all the breakpoints found by the previous authors. In cases in which we found additional breakpoints, we describe them below, but otherwise we omit the plots for brevity.

### HIV-1 CRF01_AE/B from Malaysia

**Figure 4:**
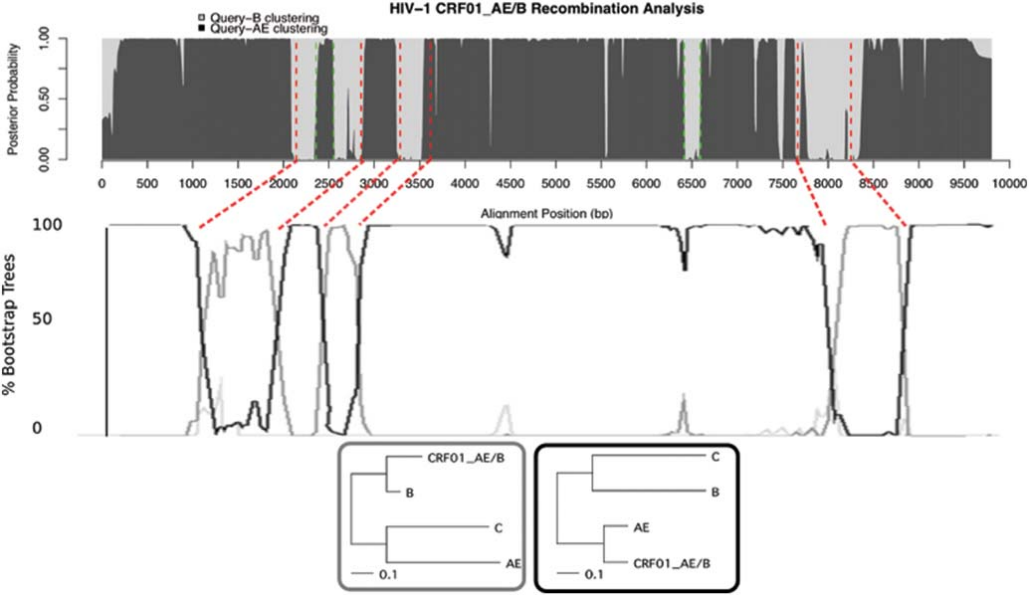
The top figure shows our analysis of the strain CRF01_AE/B Malaysian HIV-1 with our recombination phylo-HMM. We recover 6 previously predicted recombination breakpoints (red), and predict new regions in 6415–6594 and 2360–2553 (green). The grey and black regions correspond to posterior probabilities of the trees shown in the lowest figure. Previous bootscanning analysis of the same data is shown in the middle figure [18]. Since this previous analysis involved removing gaps from the alignment, we provide approximate mappings from our predictions to theirs, as the red dashed lines between the two figures. They provided precise breakpoint locations in [18] based on consensus HXB2 strain, which we plot in our figure as the vertical red lines. Note the spike in their plot that appears in our plot around 6500 as a recombinant region. The trees in the lowest figure were those trained as hidden states in our HMM; the black state clearly shows the query strain clustering with CRF_AE, whereas the gray tree shows a closer relationship with subtype B, in accordance with the previous findings.

Figure 4 depicts our results on a new Malaysian HIV strain previously analyzed by Lau et al. [18]. We recover all six of the breakpoints inferred by the original authors, who used a SimPlot/Bootscanning approach, and also we find two new breakpoints whose significance appears equal to those found previously. In Figure 4, we show for comparison the results from bootscanning, which Lau et al. used for their inference of recombination breakpoints. Lau et al. provided precise breakpoint positions, and these are plotted in our diagram as red dashed lines. Since bootscanning typically removes gaps from multiple alignments before analysis, the breakpoint positions do not align with Lau et al.'s plot very well, and we provide rough mapping between plots. All six of their breakpoint predictions are well-represented in our analysis. Note the ‘spike’ in likelihood at around nt 5800 in Lau et al's plot. This region registered as strongly recombinant in our analysis, depicted as the grey region in region nt 6415–6594. Lau et al.'s characterization of the 1500 to 2000 region ( 2141 to 2856 in ours ) is marked somewhat by uncertainty in the optimal tree topology; their “% trees” line wavers and is never very close to 100%, in contrast to their inference of region 3000 to 5500, where the line remains constant and close to 100%. This uncertainty suggests that there may be additional recombination points within that region, as is more conclusively shown in our diagram. We venture that the region between nt 2141 and 2856 can be further partitioned by two more breakpoints, at nt 2360 and 2553, shown in Figure 4.

When using bootscanning, there is a lower limit to the size of the recombinant region that can be found, which depends closely on the size of the sliding window. The 3283–3617 region, at just over 300 nt, is clearly found, but smaller regions registered only as spikes or showed uncertainty of the region. Our method is probabilistic, and instead of defining sharp partitions of the alignment, we allow the parameter training to gradually decide which regions to use to train different trees. In our analyses, we consistently found that our program is able to find small recombinant regions better than others' methods. In this case, as the posterior probabilities become more certain of the alignment partitioning, each of the grey regions contributes its information in updating the grey tree. If a sliding window was being passed over the alignment, each region would have to ‘fend for itself’ in conveying its phylogenetic signal, and small regions would go undetected. Because we use information from the entire alignment during tree-optimization and sum over all possible tree-column assignments, our approach is computationally more expensive but allows collaboration between small recombinant regions, and, consequently, improved detection.

### HIV-1 A/C Recombinant 95IN21301 from India

We examined data from the original SimPlot paper by Lole et al. In this work, the authors test five newly-sequence HIV-1 strains from India, and find one of them to be recombinant [10]. We examine this strain and confirm all five of their breakpoints and offer one new prediction. SimPlot detects mosaic strains by plotting the similarity of a query strain to other subtype reference strains. The similarity is computed within a sliding window of predefined size, according to various criteria (eg Hamming distance, Jukes-Cantor distance, etc). The result is a visualization of the closest relative of different regions of the query sequence. This is similar in effect to bootscanning distance-based phylogenetic methods (eg Neighbor-joining), and suffers from many of the same pitfalls. For example, in their whole-genome analysis of strain 95IN21301, Lole et al. used a window size of 600 bp, severely limiting the resolution of recombination detection. They conclusively found breakpoints around nt 6400 and 9500 (since gaps were removed, it is difficult to determine exact breakpoint predictions from their plot alone). They then did a second, finer-scale analysis with window size 200 bp on just the *env* and *nef* genes which were suspected to be recombinant. Within each of these single-gene regions they found an additional breakpoint in which the query sequence more closely represented subtype C.

**Figure 5:**
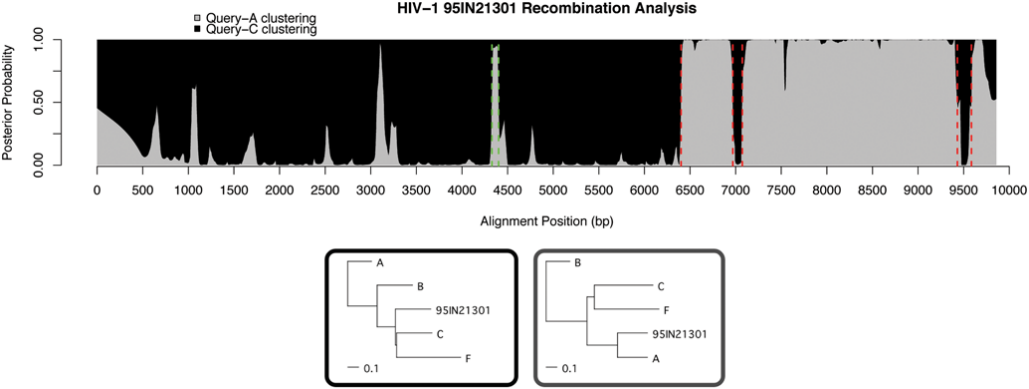
Analysis of A/C Indian HIV-1 recombinant strain 95IN21301. In the original paper [10], gaps were stripped and so mapping predictions to our plot is difficult. Instead, we show our confirmations in red, which correspond closely to the predictions seen in Figures 1 and 2 of [10]. Our new prediction of region 4328–4401 is shown in green. Trees trained as hidden HMM states are shown underneath, with their colored boxes corresponding to the colors in the plot, which in turn denote posterior probabilities of hidden states. Note that in the black tree the query sequence doesn't cluster with C, but the branch length from the (C,F) clade to the query strain is effectively zero, indicating a star-like topology in these areas.

In our analysis, we confirmed all five of these breakpoints by using our method (again, gap-stripping made exact comparison somewhat limited), and our result is shown in Figure 5; breakpoints previously found are in red, and new predictions in green. Since we do not have to specify a window and use instead a probabilistic weighting scheme, we are able to detect large regions (eg the break at position 6402) just as well as shorter regions (eg 6969–7073, 9431–9585). Furthermore, the method uses information from the entire alignment, rather than partitioning it by windows. In this case, it's possible that attempting to train a phylogenetic tree 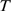 on the region 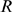 between nt 6969 and 7073 wouldn't have yielded conclusive results. If region 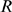 has high posterior probability of being generated by the ‘black’ tree that is dominant from positions 1–6402, the following M-Step will incorporate more counts from region 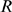, and so when region 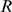 is examined, the inference algorithm recognizes that these columns ‘fit’ perfectly with the black topology, which corresponds to having high emission probabilities from the black HMM state. Also, a short 83 bp region is found supporting grey topology, in which 95IN21301 clusters with subtype A. This region is short, and its posterior probability never reaches 1, but a neighbor-joining tree on this section, 4328–4401 supports this clustering. In this way, our method is able to take into account information from the entire alignment, rather than defining a rigid window which can skip over small recombinant regions.

### Three HIV-1 BF recombinants from Brazil

We considered data from Filho et al. [19]. Their data was composed of 10 newly sequenced strains from Brazil determined to have varying levels and structure of mosaicism, as determined by bootscan analysis. We confirm their predictions (from Figure 2 of [19]) in strains PM12313, BREPM11871, and BREPM16704 and we find several more small recombinant regions. Each of the new recombinant regions we find share breakpoints with other strains we analyzed as well as strain CRF12_BF [20], suggesting they could be hotspots for recombination activity.

**Figure 6:**
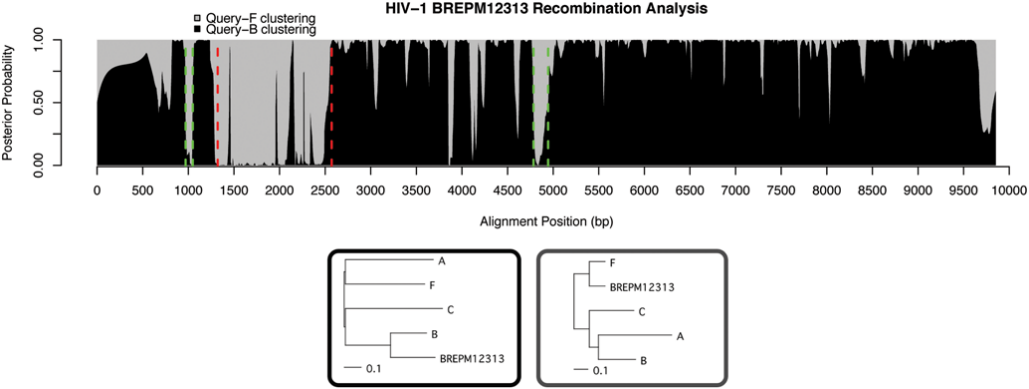
Brazilian strain BREPM12313. We confirm Filho et al.'s breakpoints near 1322 and 2571 (red), and predict new recombinant regions in nt 4784–4945 as well as 970–1049 (green). The second of these is short, but present in some form in all three strains analyzed here. The spike at 3851–3909 is even shorter and is not represented in the other two species, leading us to not predict it as a likely recombinant region. Trees trained in hidden states are shown below the plot.

As seen in Figure 6, strain BREPM12313 showed a clear recombinant region from nt 1322–2571, previously characterized by Filho, et al. Also, a region around 4700–5000 showed some evidence of recombination, having the same topology as 1322–2571. As this region's posterior probability is more ‘spike-shaped,’ rather than having sharp borders between colors, it is difficult to say whether or not it is an ambiguous region or a genuine recombinant. It does share one crossover point with strain BREPM11871, giving it somewhat more credibility. Performing neighbor-joining on nt 4784–4945 (eg positions where posterior probability is higher for grey) showed BREPM12313 clustering with subtype F. At the end of the genome, another possible recombinant region is seen, at around 9700. This region includes only gaps for the query sequence, and thus the inference is not reliable. Our method treats gaps as missing information, and when they are present in small numbers reliability is not affected, but in places like this where only gaps are present it can hinder the tree-inference.

**Figure 7 pcbi-1000318-g007:**
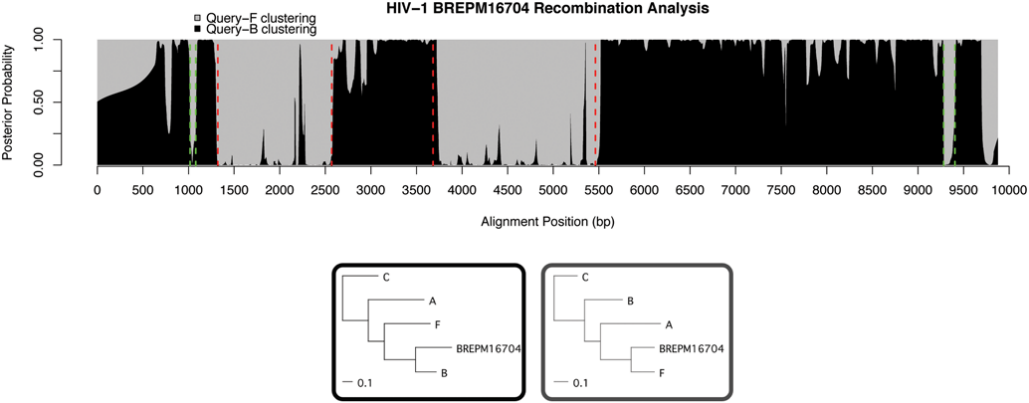
Brazilian strain BREPM16704. We confirm breakpoints near 1322, 2571, and 5462 (red) and predict recombinations in 9281–9405 and 1017–1085 (green). Trees trained in hidden states are shown below the plot.

Strain BREPM16704 was previously predicted to have four breakpoints, which we recovered with remarkably high posterior probabilities for the tree-states. Figure 7 shows our results with previous predictions in red. A new region, at 9281–9405, shows high posterior probability and is common to BREPM11871 and CRF12BF [20], making a strong case for a recombination hotspot.

**Figure 8:**
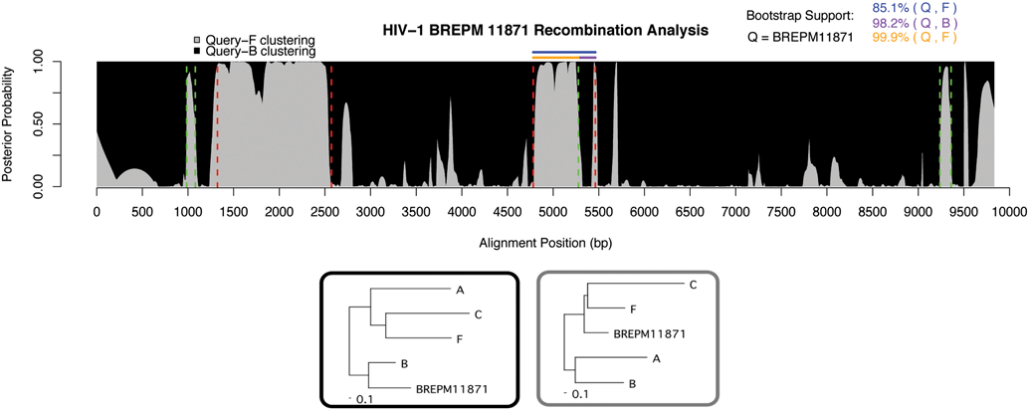
Brazilian strain BREPM11871. Confirmation of breakpoints 1322 and 2571, and 4782 (red dashed lines). We predict a region common to BREPM16704 at 9238–9361 (green). Also, the breakpoint previously estimated at 5462 (red) we propose to be at 5277 (green dashed line). In support of this, we provide bootstrapping values (1000 replicates) for the 3 different regions, indicated by horizontal colored lines above the plot. Our prediction (orange) carries the highest value, 99.9%, whereas the previous (blue) is only 85.1%, since it includes a region (purple) that strongly supports BREPM11871 clustering with subtype B, with value 98.2%. The small region at 985–1080 is difficult to confidently categorize, but its high posterior probability for clustering with F and its agreement with the other two strains lead us to suspect a recombination. Trees trained in hidden states are shown below the plot.

In strain BREPM11871, all four breakpoints predicted by Filho et al. were found, as well as a new crossover region, common to BREPM 16704, at 9238–9361 (shown in green in Figure 8). The break previously described at nt 5462 bp was predicted by our method to be at 5277. To determine the more likely crossover point, we performed 1000 bootstrapping trials on each of the following regions: 4782–5277 (our prediction), 4782–5462 (Filho et al.'s prediction), and 5277–5462 (the disputed region). We found that the 5277–5462 region strongly supported BREPM11871 clustering with subtype B, with 98.2% bootstrap support. Moreover, bootstrap support for query-F clustering appears higher for our predicted region (99.9%) than the previous prediction (85.1%). We conclude that our algorithm often outperforms previous methods in accurately determining recombination breakpoints.

## Discussion

Recombination is an important force driving genome evolution, and in several cases it is the primary force for diversity. As such, methods which can detect and characterize recombination events are crucial to the successful utilization of new sequence data. On the single-gene level, recombination has been shown, in at least one case, to be able to confer antibiotic resistance [17]. It could be possible that inter-subtype recombination conferring drug resistance is a common phenomenon, which could be investigated using our methods. On the multiple-gene scale, *Chlamydia trachomatis* has been shown to undergo frequent inter-subtype recombination resulting in mosaic genomes [4] which complicate subtype definition and classification. On the genome scale, HIV-1 has long been known to participate in recombination leading to several identified circulating recombinant forms (CRFs). For these clinically relevant pathogens, accurate detection of recombination following introgression is important not only to guide disease treatment methods, but also for tracing the epidemiological history of the virus. In this work we present a novel method for recombination detection which we believe to be more sensitive, flexible, and robust in the aforementioned evolutionary scenario. We combine two long-standing concepts, phylogenetics and hidden Markov models, in a maximum-likelihood framework to model topology changes over an alignment of related sequences.

We present a training scheme which attempts to solve the two problems embedded within recombination detection simultaneously. We model evolution probabilistically with a continuous-time Markov chain which directs the likelihood-based tree construction algorithm [21]. Furthermore, our alignment-partitioning is handled with posterior probabilities which take into account each hidden state tree. By summing over all possible tree-column assignments and not using sharp window cutoffs, we are able to perform more precise breakpoint determination. We can adjust the specificity and sensitivity of the model with the transition matrix of the HMM, which dictates how much of a likelihood change should cause the model inference to change states. We believe this *likelihood* comparison to be superior to adjusting the size of the window because it enables distant sections of the alignment to combine their phylogenetic signal in training a hidden state of the HMM.

## Methods

The goal of this study is to start with a multiple alignment of sequence data and find positions where recombination events have occurred. This is done by recovering a set of phylogenetic trees and a map that assigns a tree to each column. The points at which neighboring columns have different tree assignments will indicate possible locations of recombination events in evolutionary history.

### EM for Recombination-HMM

We use a hidden Markov model with tree topologies as hidden states and alignment columns as observed states. The usual method to train HMM parameters is by the specialization of the EM algorithm known as the Baum-Welch algorithm. Our transitions can be optimized in the usual way, but the emissions are more difficult since their likelihood is governed by the tree topologies in the hidden states, which are not easily optimized. For this problem, we employ an EM method for trees, developed by Friedman et al. [21], within our M-step for emission probabilities. Their phylogenetic inference algorithm is implemented with such improvements as simulated annealing and noise injection in SEMPHY, available from their website at http://compbio.cs.huji.ac.il/semphy/. We instead implemented our own ‘bare-bones’ version of this algorithm, without these improvements. To progressively assign columns to trees, at each M-step, we weight the expected counts of the tree-EM by the posterior counts of the phylo-HMM. Intuitively, this guides the tree-maximization by providing comparatively more information from regions which fit a particular tree. We give a high-level description of our method here and more detail is provided in Text S1. Figure 10 gives a graphical representation of the overall task-flow. After running our program on the alignment data to estimate parameters, a state path through the HMM is computed from the final matrix of posterior probabilities. We would like this path to represent a balance between being biologically reasonable and highly probable under our model. Thus, we propose maximizing the sum of posterior state probabilities subject to a length cutoff by the following method.

Let 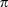 be a path of length 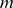 through the state space of the HMM (discounting start and end states), emitting the first 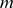 columns of the alignment, with one state per column. Let 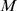 be the total number of columns in the alignment. We say that the state path is *partial* if 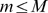, and *complete* if 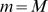.

Let 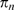 denote the 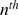 state in path 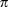. The *score* of path 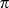 is defined as 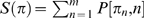 where 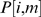 is the posterior probability that column 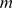 was emitted by state 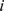.

We say a state path 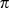 is *valid* if all its breakpoints are more than 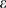 apart. That is, there exist no 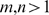 with 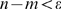 such that 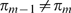 and 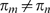.

Finding the maximal valid 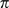 is solved by a simple dynamic programming procedure, outlined in Text S1.

**Figure 9:**
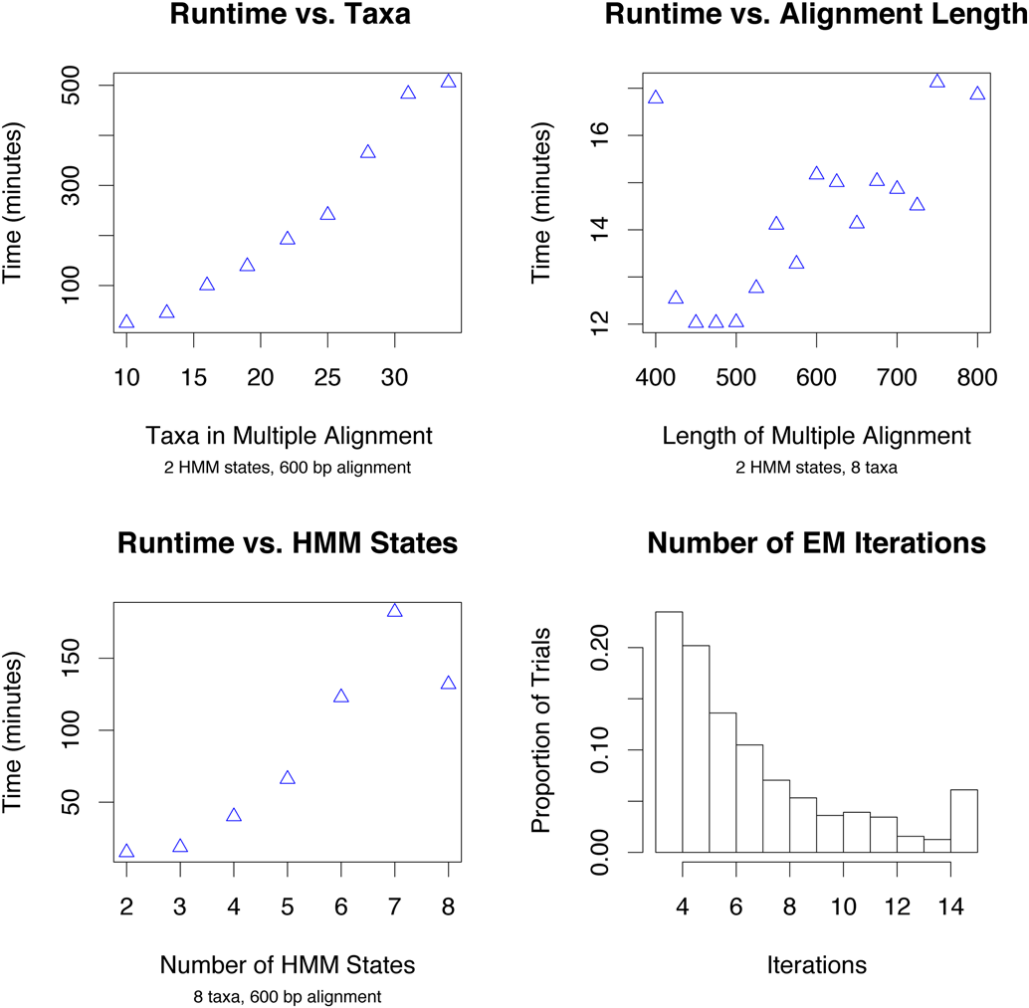
Resource use of the algorithm increases with model complexity. The algorithm converges in a reasonable number of EM steps, as seen in the lower right plot. We observed no dependence of iterations to convergence and the model complexity, and so the lower right histogram represents data concatenated from all simulation trials. The final bar in the histogram represents the proportion of trials which took 14 or more iterations to converge.

Since the EM algorithm has a tendency to converge to local likelihood maxima which are not global maxima, especially when initialized randomly, we ran the algorithm several times for each dataset, took the highest-likelihood result for the set of trials, and performed the above posterior decoding on the final distribution. We show plots of our program's performance when various aspects of the model and input alignment are varied in Figure 9.

**Figure 10:**
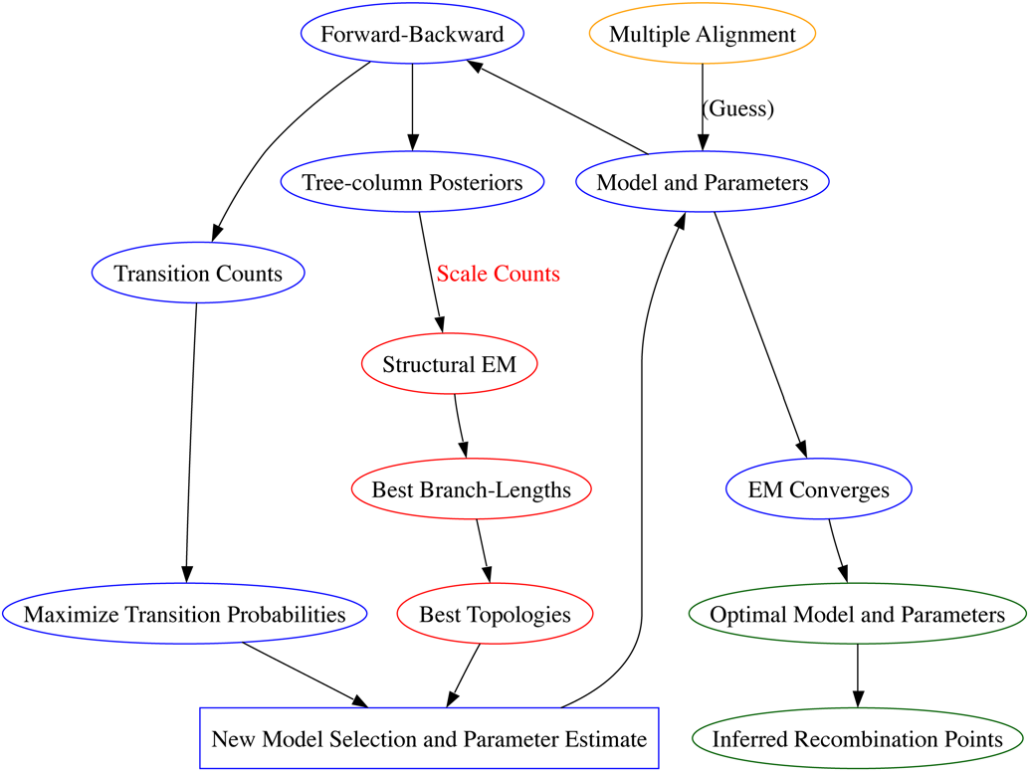
Phylo-HMM training algorithm. Input Alignment ⇒ Model Selection/Parameter Estimation ⇒ Recombination Inference.

### Algorithm: Recombination Phylo-HMM Training

Input: An alignment 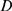, an integer 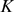 specifying number of trees, a guess at the true tree topologies and transition matrix. In practice, we initialized the transition matrix to have values of .999 on the diagonal, and split the remaining .001 among the remaining columns. Transition probabilities are trained in the normal Baum-Welch manner. We noted that inference was relatively robust to the initial value of this parameter.
Output: A proposed MLE of 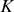 phylogenetic trees and the transition matrix. This determines a posterior state distribution, from which we deduce breakpoints.
E-Step 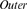: Calculate the Forward, Backward, and posterior probability arrays in the usual manner for HMMs, as in [22]. The emission probabilities for each tree-state are computed by Felsensteins Pruning algorithm [23]. Use the Forward and Backward arrays to compute the expected transitions between hidden states.
M-Step 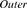:
  1. Maximize the transition probabilities to obtain a new estimate of the transition matrix.
  2. Find a new set of trees, using model selection with Structural EM:
E-Step 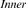: For all 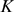 trees, compute expected counts of hidden data 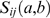 (see below). Scale counts for tree 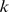 from column 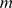 by the posterior probability that column 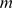 was emitted from state/tree 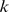.
M-Step 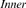: Increase the likelihood of each tree topology with Structural EM (see below) [21].

The hidden data for each tree are defined as 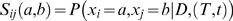, the number of transitions from nucleotide 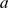 to nucleotide 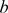 from node 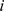 to node 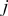, for all pairs of nodes. For neighboring nodes, this can be computed by Elston and Stewart's Peeling algorithm [24], and for non-neighboring nodes, exact computation requires a dynamic programming routine described in the original Structural EM paper [21]. In this work, the authors showed that the likelihood contribution of an edge between two nodes can be summarized as a function of this count 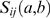.

If we arrange these summaries in a weight matrix 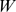 in which 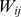 represents the expected likelihood contribution resulting from placing an edge from node 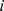 to node 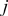, it is easy to see that the maximum expected likelihood tree will be the topology which maximizes the sum of its edge scores. Finding such a topology is trivial if we do not require that the tree is binary, for instance by maximum spanning tree algorithms. This (possibly non-binary) tree is then transformed to a binary tree by operations which do not alter the tree's likelihood. In this way, Structural EM allows for iteratively constructing higher likelihood trees by choosing the next tree which maximizes the expected likelihood based on the current tree. The reader is referred to Text S1 and the original Structural EM paper [21] for more detailed discussions of this algorithm which is crucial to our method.

In our methods, instead of allowing Structural EM to converge, we allow two iterations using the same set of transition and posterior probabilities, as a heuristic substitute for finding the true hidden tree topologies.

### Possible Extensions

We outline here a number of extensions which could grow directly from this work. One of the strengths of the method is its generality and flexibility, and so we believe it is ideally suited for continued development.

- **Sequence Evolution Model** Currently we model gaps as missing information (e.g., summing over possible values). This is not realistic and may hinder phylogenetic reconstruction, and consequently recombination inference. The simplest possible next step is to treat a gap as a ‘5th nucleotide.’ While this assumes independence among inserted and deleted residues, it has been shown to aid phylogenetic reconstructions more than treating gaps as missing characters [25]. Our code uses the HKY85 substitution model, whose matrix exponential is solved in closed form. A more general rate matrix diagonalization and exponentiation is currently only implemented in the Python prototype of our core dynamic program, which we find to be too slow (the experiments reported in this paper used a core dynamic programming algorithm implemented in C, for speed). This is, however, purely a technical issue, and modeling gaps is entirely feasible. Similar elaborations of the substitution model, such as codon evolution (if the region of interest was protein-coding) or an extra hidden state determining coding and non-coding regions, might provide more accurate modeling of large genome-scale alignments.
- **ARG-like trees and** 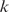 The method currently does not restrict the 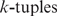 of trees produced at each iteration. As [26] point out, not all groups of trees can fit together to produce an ARG. Restricting the tree groups would give a more conclusive answer to the epidemiological recombination question, and may even be helpful in informing the tree selection heuristic. One can imagine a simple extension to our method which attempts to learn 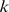 as well as producing consistent trees:

At each training iteration:

- If the trees maximized in each hidden state are not consistent:

* First, find a set of trees in the usual manner, without regard to whether they are consistent. Then, find the best-scoring set of trees which are consistent. This is computationally intensive but not intractable, since we can enumerate ordered spanning trees for each of our hidden states from our E-Step. Once this set has been found, if the likelihood difference between the inconsistent and consistent set is deemed acceptable, accept the trees and begin a new training iteration.
* Otherwise, if this likelihood penalty is deemed to large, we recognize that the current 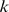 is inadquate to describe the data, and so we add a new hidden state to the model, and continue training.

A simpler way of estimating 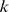 would be to run a coarser heuristic method (e.g., SimPlot) and seed the HMM with the number of states that it finds.

### Sequence Data

All sequence data used in this study was downloaded from public databases (GenBank and LANL HIV Database). The sequences were aligned with MUSCLE [27] with the default parameters. Gaps in the alignments were treated as missing information. Bootstrap analyses were performed with CLUSTAL W [28] with 1000 replicates and the default parameters. The GenBank identifiers for sequences used are as follows, grouped by figure: Figure 3: argF: X64860, X64866, X64869, X64873; penA: X59624–X59635; Figure 5: AF067158, AB253429, AF067159, M17451, AF005494; Figure 4: AB032740, AB023804, AY713408, EF495062; Figures 6–8: AF286228, AF005494, AY173956, AB098332, AY173956, DQ085867, DQ085876, DQ085872.

The source code for our programs, though still being developed, is available upon request or through CVS. For documentation, contact, and download information See http://biowiki.org/RecHMM

## Supporting Information

Text S1 Results on simulated data; detailed descriptions of training and decoding algorithms
(8.74 MB PDF)
