# Supplementary material for: Accurate Detection of Recombinant Breakpoints in Whole-Genome Alignments
Source: PLoS Comput Biol. 2009 Mar 20;5(3):e1000318. doi: 10.1371/journal.pcbi.1000318 (PMC2651022; doi:10.1371/journal.pcbi.1000318)
Supplement: Text S1 — Results on simulated data; detailed descriptions of training and decoding algorithms (8.74 MB PDF) [file pcbi.1000318.s001.pdf]

Supplementary Information

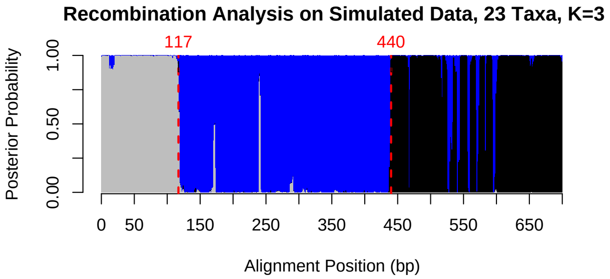

Figure 1: Synthetic alignment of size 700 × 23, with two breakpoints (shown in red). Both breakpoints are topology-changing and both are detected with great accuracy, although there is some uncertainty in region 530-600.

Results, Simulated Data

In analyzing actual biological data, there is no easy way to verify the predicted recombination breakpoints, so to investigate the accuracy, reliability, and limits of our method, we applied our algorithms to data simulated by **Recodon**, [1]. We produced several synthetic datasets of varying lengths, number of taxa, number of recombination events, and structure of marginal trees. The general simulation parameters used were the following: recombination rate: 2×10^{-8} , mutation rate: 3×10^{-4}, {A,C,G,T} frequencies: {.3 .2 .2 .3}, transition/transversion ratio: 2.0.

Taxa number presented more of a limitation in actual data than in simulated data; on synthetic datasets we obtained conclusive results on alignments with up to 30 sequences. This is most likely due to the simplicity of the generated data; when modeling evolution on real data, simple Markov chain approaches greatly simplify the process, and we sum out gaps as missing information, whereas in the simulated data there were no gaps. Our simulated analyses were very accurate, usually if a breakpoint was detected, it was accurate within 10 positions. With marginal trees which differed only in subtle ways, such as branch-length or deep branching patterns, our programs rarely detected changes, whereas with changes at the leaves detection was near very strong. More HMM states than recombinant regions wasn't problematic, whereas having too few HMM states led to inaccurate or missed breakpoint predictions. We believe this to be the limiting factor in our method; it was uncommon to see a decisive model which employed more than 4 trees.

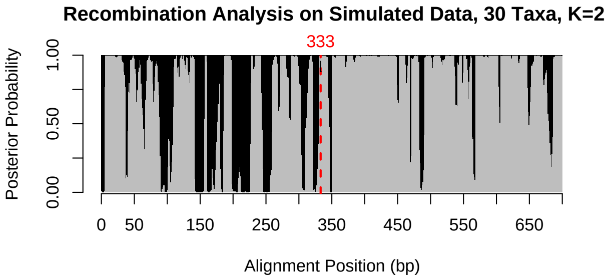

Figure 2: Synthetic alignment of size 700 × 30, with one breakpoint (red). The entire posterior distribution is uncertain and no inference can be made. There is somewhat less 'noise' in the signal after position 333, but other than this there is little to be learned from the results.

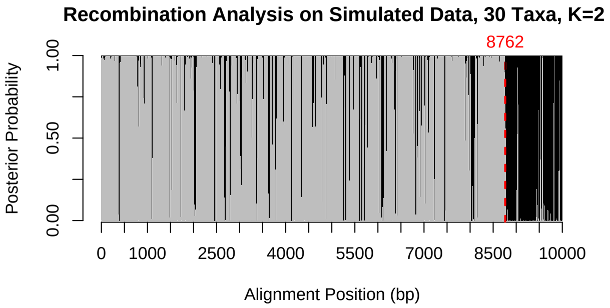

Figure 3: Synthetic alignment of size 10,000 × 30, with one breakpoint (shown in red). The recombination point at 8762 is found, despite having to compare trees with 30 taxa. Longer alignments tend to have a clearer/stronger phylogenetic signal, which may be why this recombination was detected while the breakpoint in Figure 2 was not.

Many Taxa Intuitively, our methods will become less powerful the more taxa are included in the alignment, since phylogenetic tree estimation becomes more and more difficult. Note that there are many ‘spikes’ in the posterior distribution in positions 500-600 Figure 1, signifying uncertainty of inference, and in Figure 2 the posteriors are uninformative. Still, the program is often able to do quite well even with many taxa, such as in Figure 3, where the posteriors are somewhat noisy but it is still clear where the change happens. The ability to include many taxa in a recombination analysis is very valuable, especially when the involved species are unknown. The reason for the decay in inference is that a group of phylogenetic trees with many taxa will have very similar likelihoods, and thus differentiating between them in order to partition an alignment is a difficult task.

Ancient Recombination In situations where recombination has occurred long ago, ‘deep’ in the phylogenetic tree, an entire subtree is transferred. This type of recombination is typically not detected very well by our method, since the ideal phylogenetic trees differ in their branching near the root, as opposed to near the leaves. This leads to the inference algorithm modeling the two regions by a single topology, since the likelihoods of the actual different trees is relatively similar. Practically speaking, this is not as clinically important as detecting recent recombinations. In a typical inter-subtype recombination analysis, the reference strains are taken to be ‘pure’ in their subtype, and the search for recombination only concerns the present-day clinical species. In the case when a few taxa in a large tree (i.e. 2 taxa in a 30-taxa tree) are transferred, this is usually detected. This could arise when analyzing a group of clinical isolates against a set of reference strains where two of the isolates had undergone similar recombination events. Figure 4 and 5 show an example where an ancient recombination is missed and a recent leaf-changing topology change is clearly detected.

Too many HMM states When the number of tree-states is larger than the number of distinct regions

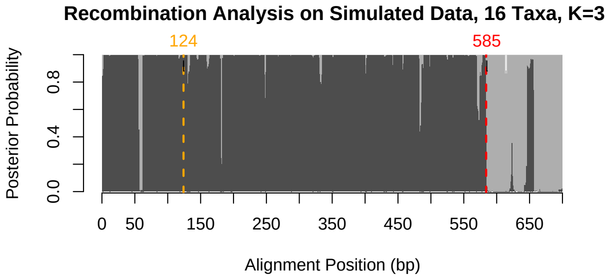

Figure 4: Synthetic alignment of size 700 $\times$ 16, with two breakpoints. Breakpoint at 124 (orange) changes the topology near the root of the tree, whereas breakpoint 585 (red) changes tree topology near the leaves (see topologies in Figure 5).

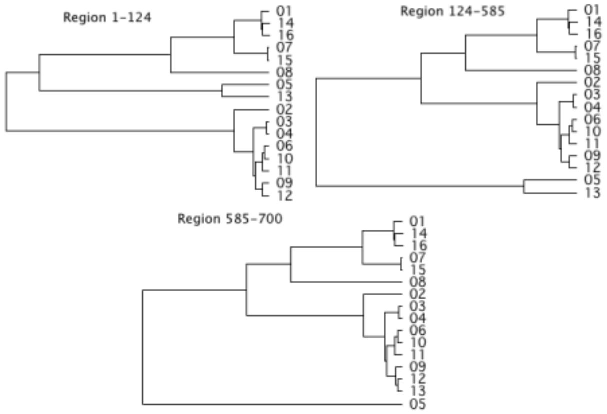

Figure 5: Tree topologies for different regions in Figure 4. The transformation between trees (1-124) and (125-585) involves a subtree transfer deep in the tree and so the breakpoint at position 124 goes undetected. In contrast, the difference between (124-585) and (585-700) involves transferring leaf 13, which is more readily detectable.

in the alignment, this is typically not a problem, as seen in Figure 6. One of the trees either remains at low posterior probability or only appears for very small regions. In this way, the training algorithm recognizes that it has an 'extra' tree that it doesn't need to accurately model the data. In some cases, however, this extra state can be employed to model a more highly-diverged region which has a different optimal topology, but which is not actually a recombinant region. In Figure 6, the blue tree topology remains at low probability except for two very short regions, and the breakpoint is still well-predicted.

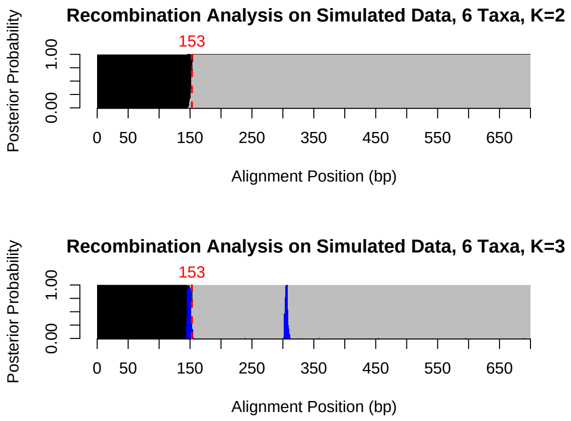

Figure 6: When K=3, but there are only 2 recombinant regions, the third tree is unused except for a very short region near the crossover point, and a spike near bp 300.

Too few HMM states When the number of recombinant regions is more than the number of tree-states in the HMM, this has mixed effects. In Figures 7 and 8, the dataset actually had 5 breakpoints, and each region was topologically distinct, but only 4 resp. 6 trees were used to train the model. Sometimes topology shifts are detected, even if the this region does not have its own tree to train, but in general the model has a difficult time decisively detecting breakpoints. Adding more states, as Figure 8 attempts, does not appear to enable detecting more regions, leading us to believe that number of distinct-topology regions of the alignment is the limiting factor in our methods. We are investigating more robust initialization and training methods which might enable training many more states. The requirement that the different tree topologies fit together to form an ARG somewhat constrains the trees to look rather similar. If we relax this requirement, trees can differ by more than 1 subtree prune and regraft move (the operation of detaching an edge and reattaching it somewhere else in the tree), which leads to more radically distinct topologies. When we simulate alignments with these sorts of topologies, we are more readily able to detect 5 or more regions, as shown in Figure 9.

Small regions Window-sliding methods typically perform badly in detecting small regions (eg less than 200 bp), whereas we are able to reliably detect small regions if they have distant supporting regions, and often even if they don't. In our method, all the information in the alignment is combined to construct trees, allowing distant but similar regions to collaborate in training trees which improves detection. If the short regions have a topology in common with a longer region in the alignment, they are typically detected very well, because their tree was mostly trained elsewhere in the alignment. If they represent a unique topology, it is difficult to construct a tree on such a small region, and inference is limited. If there are several small regions of a unique topology, however, they can combine their information to make detection more feasible. Figure 10 shows results on an alignment with four small recombination regions which are all detected accurately. The small regions at 1000-1100 and 1600-1700 pool together their phylogenetic signal in order to train the black tree topology.

Methods in Detail

Definition: The *Ancestral Recombination Graph* (ARG) is a labeled directed acyclic graph in which nodes correspond to species and edges correspond to evo-

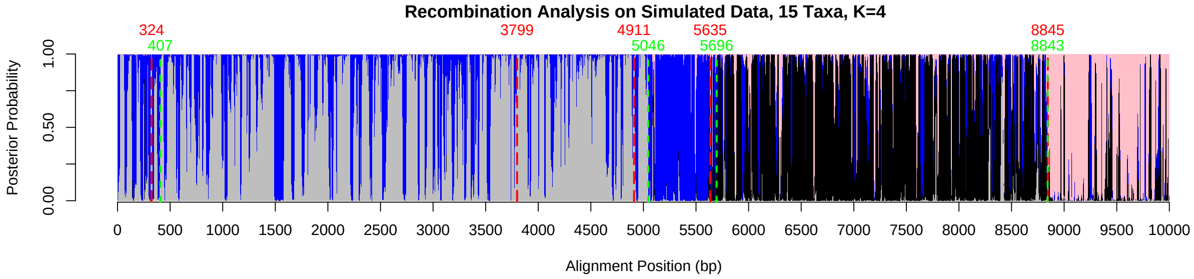

Figure 7: When K=4, but there are actually 6 recombinant regions, inference is hampered. There are too many distinct topologies to train and the model is unable to accurately partition the alignment, leading to one breakpoint undetected and one (4911) predicted poorly. The predicted breakpoints (green) which are close to the actual simulated breakpoints (red) are labeled above the alignment. Those regions which are predicted have somewhat inconclusive posterior distributions, making inference difficult.

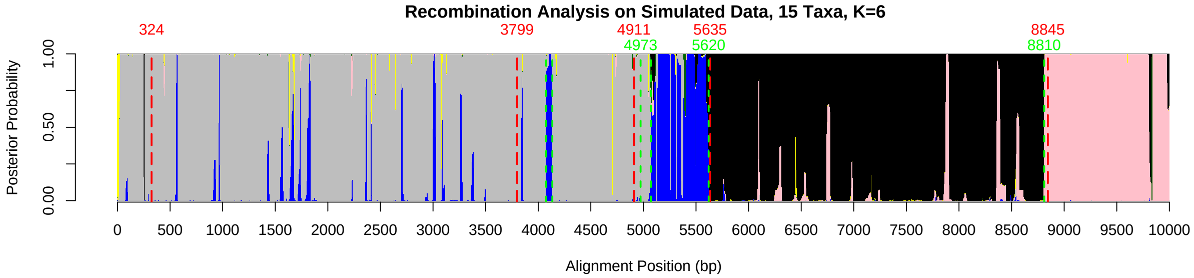

Figure 8: Using the same data as Figure 7, but setting K=6, we still get 2/5 breakpoints undetected. The training algorithm has a difficult time employing more than 4 trees in the HMM. In contrast to Figure 7, the posteriors are quite decisive and the predicted breakpoints are reasonably accurate.

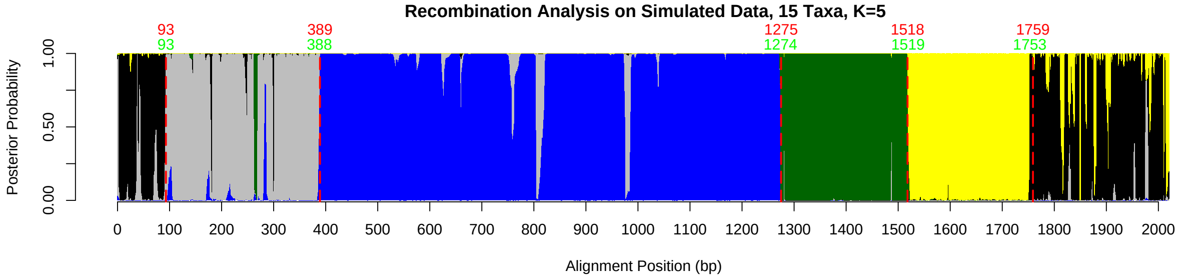

Figure 9: Using data generated with arbitrary tree topologies, rather than ones that fit together to form an ARG, we are able to train 5 distinct trees, and locate the breakpoints with great accuracy. Relaxing the ARG requirement allows for radically different tree topologies which are easier for the program to detect. This alignment contained 6 distinct regions, but the first and last were somewhat similar, and here they are detected as being the same. Since these two are not neighboring, all breakpoints are detected well.

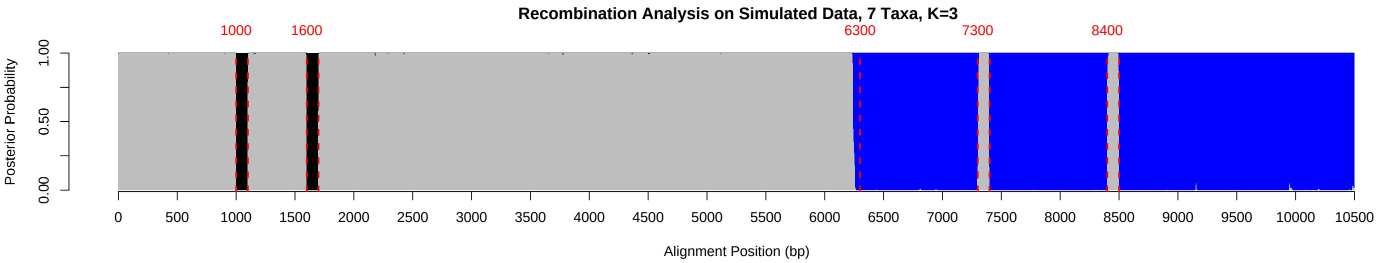

Figure 10: Small recombinant regions. In addition to the main breakpoint at position 6300, this alignment contains four small crossover regions of size 100 bp (one label per small region, for clarity). The regions at 7300 and 8400 are of the same topology as the large grey region in the first half of the alignment, and so they are detected remarkably well (all four breaks accurate within 4 positions). The regions at 1000-1100 and 1600-1700 contain their own topology which appears nowhere else in the alignment, and so their information is contributed only to the black tree. This allows the black tree to be trained especially for those regions, leading to very close detection (accurate within 2 bp).

lutionary relationships. Leaf nodes are assumed to be present-day species, and are taken to be observed, whereas ancestral nodes are hidden data. Edges can be solid or dashed, where a solid line indicates that all of the child's genetic material evolved directly from the parent's. A leaf under a dashed line represents a recombinant child, in which some of their sequence material came from one parent and some from the other. If the position of recombination is assumed to be known, then an ARG is able to convey all evolutionary information of a group of present-day taxa [3]. In contrast to a phylogenetic *tree*, this *graph* contains nodes that have more than one parent, in this case, node 3. As node 4 and node 5 *recombined* or *crossed over* to form node 3, part of 3's sequence is descended from node 4 and the other is descended from node 5.

Definition: A *Marginal Tree* is a phylogenetic tree arising from an ARG by way of realizing a set of dotted edges such that each leaf node has exactly one parent, and internal nodes without children are removed. It is assumed that each position of the alignment can be described by single marginal tree.

At any given point in species 3's genome, its ancestry depends on which of the dotted edges was realized above it. In the ARG shown in Figure **??**, there are two possibilities, and thus there are two *marginal trees* that exist within the ARG. These trees are shown in Figure **??**, with realized edges shown as solid and non-realized edges in gray.

Parameters and Notation Here we describe the notation we use throughout this paper and the parameters we wish to learn from the data. In general we follow the notation of [2] and [6], extending it when necessary. While at present we must specify K, the number of trees in the model, an eventual goal is to be able to learn K from the data, allowing the training routine find the best number of states.

- D is a multiple sequence alignment, taken as input. This is an array of N biological sequences, each of length M in which each column is assumed to have evolved from a single ancestral character [6], except when gaps are present. Gaps are treated as missing information. We use the terms 'position' and 'column' interchangeably as our model generates one column per position.

- $\{(T,t)_k\}$ is a set of phylogenetic tree topologies T and branch-length vectors t for each of the K hidden states. Strictly speaking, $\{(T,t)_k\}$ is not a *parameter* of our model, but it actually is part of the architecture of the model, since it models how sequences in the multiple alignment came to their present state via a certain evolutionary pathway. This is a subtle point since $\{(T,t)_k\}$ dictates θ^e, which is a parameter matrix of the HMM. Thus, whenever we re-

estimate $\{(T,t)_k\}$, we are performing model selection as opposed to parameter estimation.

- θ^s is a stochastic matrix of size $K \times K$ in which the k,l entry indicates the probability of transitioning from tree k to tree l.

- θ^e is a stochastic matrix of size $K \times M$ where N is the number of sequences in the multiple alignment and M is the length of the alignment. $\theta^e_{k,m}$ represents the probability that tree k emitted column m of the alignment. We recognize that our choice of tree topologies defines our emission matrix in the following way:

$$\theta^e_{k,m} = P(D_m|(T,t)_k) \tag{1}$$

  Where D_m is the mth column of the alignment data and $P(D_m|T_k,t_k)$ is the *marginal likelihood* of tree k producing D_m, which can be computed by Felsenstein's pruning algorithm [5].

- For simplicity and clarity of notation, we will henceforth refer to the set $\{(T,t)_k\}$, θ^e, and θ^s as θ or the "parameter set." We keep in mind that $\{(T,t)_k\}$ is actually part of the model structure, but since we are updating these three objects at each iteration, it makes intuitive sense to refer to them as a unit. When describing an algorithm such as EM where the parameter set is iteratively improved, $\theta^{(n)}$ refers to the parameter set at the nth iteration.

- $P(D|\theta)$ represents the likelihood of the observed alignment data under our model and a particular parameter set θ.

- With $X = \{x_1, x_2 ... x_M\}$ representing the hidden data (in this case an assignment of phylogenetic trees to columns of our alignment), $P(D,X|\theta)$ is the probability of the fully observed data under the model, given by the equation

$$P(D,X|\theta) = \theta^s_{start,x_1} \prod_{i=1}^{M} \theta^e_{x_i,D_i} \theta^s_{x_i,x_{i+1}} \tag{2}$$

  Further, the hidden and observed data are related by the property that

$$P(D|\theta) = \sum_x P(D,x|\theta) \tag{3}$$

  Where x ranges over all M^K possible sequence assignments to the hidden nodes.

- $P(x_m = k|, D, \theta)$ denotes the posterior probability of a particular state emitting a certain column, conditioned on the parameters and alignment data. For details on the computation of this quantity, see [2].

- $P_{a \to b}(t)$ provides an underlying evolutionary model specifying the probability that symbol a evolved into b under time t, for $a, b \in \Sigma, t \geq 0$, as well as a prior distribution over Σ. This is assumed to be part of the evolutionary process and in this work we do not try to refine the model as we estimate the previous parameters.

Baum-Welch Algorithm The special case of the EM algorithm applied to HMMs is known as the Baum-Welch algorithm [2]. This takes advantage of the tree-like structure of HMMs, using the sum-product algorithm to efficiently calculate expected hidden data statistics. In describing the Baum-Welch method, we introduce the following terms, following the notation of Durbin *et al* [2]:

The Forward Probability (F)

Defined as $F[m,k] = P(D_{1...m}, x_m = k|\theta)$, this represents the probability of the observed data up to and including column m, requiring that tree k produced column m. The Forward probability gives us a simple way to calculate the probability of the observed set of columns under a particular parameter set:

$$P(D|\theta) = \sum_k F[k,M]\theta^s_{k,end} \tag{4}$$

This is intuitively understood as the probability of the sequence up to M (i.e. the whole sequence), summed out over all possible states for

the last position, and then accounting for the transition into the End state.

The Backward Probability (B)

Defined as $B[m,k] = P(D_{m+1,m+2\ldots M}|x_m = k,\theta)$, this is analogous to the Forward probability, but starting at the end of the sequence. Note that here we compute a conditional as opposed to a joint probability (although both are conditioned on θ).

Posterior Probabilities

Using these Forward and Backward probability arrays, we can now calculate the *posterior probability* of a certain hidden state assignment given the observed alignment columns. We use the following property:

$$\begin{aligned} &P(x_m = k|D,\theta) \\ &= \frac{P(D, x_m = k|\theta)}{P(D|\theta)} \\ &= \frac{P(D_{1\ldots m}, x_m = k|\theta)P(D_{m+1\ldots M}|x_m = k,\theta)}{P(D|\theta)} \\ &= \frac{F[m,k]B[m,k]}{P(D|\theta)} \end{aligned} \quad (5)$$

Where $P(D|\theta)$ is computed by way of equation (4). These probabilities are useful for both inference and training. First, in searching for optimal parameters, the posteriors are what allow us to build different trees on different parts of an alignment. At each iteration we use these posterior probabilities to weight hidden data counts in the tree-optimization step. Second, once we are satisfied with the parameters and are attempting to assign trees to columns, the posteriors can be of great use. The quantity $P(x_m = k|D,\theta)$ takes into account the HMM structure, whose transition probabilities put a restrictive prior probability distribution on the number of recombination crossovers. This not only allows for a more realistic interpretation, but also allows us to adjust the sensitivity and specificity of the algorithm by controlling the entries of θ^s.

Expected Counts

The Forward and Backward calculations also enable simple computation of expected hidden event counts, namely the number of transitions $k \rightarrow l$ and emissions of string σ from state k. In this application, we only estimate the transition counts, and use Structural EM to deal with the emission maximization. To get the expected number of transitions $k \rightarrow l$, we use:

$$\begin{aligned} &E\left[\#k \rightarrow l\right] \\ &= \sum_{m=1}^{M} P(x_m = k, x_{m+1} = l|D,\theta) \\ &= \frac{F[m,k]\theta^s_{k,l}\theta^e_{l,D_{m+1}}B[m+1,l]}{P(D|\theta)} \end{aligned} \quad (6)$$

With these expected counts in hand, updating the parameter θ^s is trivial:

$$\hat{\theta}^s_{k,l} = \frac{E\left[\#k \rightarrow l\right]}{\sum_{l' \in \Sigma} E\left[\#k \rightarrow l'\right]} \quad (7)$$

Structural EM Structural EM is an iterative model selection scheme aimed at finding the most likely phylogenetic tree model for a multiple alignment [6]. It follows the prototypical EM structure in that it alternates between estimating hidden data and maximizing the model until a critical point in the likelihood surface is reached. In the E-Step, the tree topology and branch lengths are held constant and hidden statistics are estimated, namely the character transition counts between nodes. In the M-Step, a new model (a phylogenetic tree) is selected and its branch lengths chosen based on the estimated hidden statistics. This is a useful method as it makes maximum likelihood phylogenetics computationally feasible with long sequences and many taxa. Further, its estimation step allows for differential column-weighting which allows us to progressively "focus" a tree on particular alignment region.

Structural EM Steps We give a high-level description and the reader is referred to the original Structural EM paper for more detail [6]

Algorithm: Structural EM (SEM)

Input: An $N \times M$ gapless multiple alignment of biological sequences

Output: A phylogenetic tree (T, t) such that $P(D|(T, t))$ is a critical point of the likelihood surface

E-Step: Compute expected hidden data counts $S_{i,j}(a, b)$, the expected number of transitions of symbol a to symbol b from node i to node j.

M-Step $_{Branches}$: With these counts, find the branch length $\hat{t}_{i,j}$ for each (i, j) pair (not necessarily an edge in the tree) which would maximize the contribution of edge (i, j) to the tree's likelihood function.

M-Step $_{Topology}$: From this matrix of likelihood contributions, construct a maximally-scoring topology with the branch lengths chosen from the previous step.

Incorporation into Phylo-HMM Training We use SEM in training our phylo-HMM to estimate a better and better set of trees for our model at each M-step. At each iteration we perform K independent executions of SEM model selection, where during the E-Step of each one, the counts $S^{(k)}_{i,j}(a, b)$ are weighted by the posterior probabilities of the different alignment columns having been generated by a particular tree. More precisely, while maximizing the kth tree:

$$S^{(k)}_{i,j}(a, b) =$$

$$\sum_m P(x_m = k|D, \theta)P(x_i[m] = a, x_j[m] = b|D_m, T, t)$$

Where $P(x_m = k|, D, \theta)$ denotes the posterior probability of the mth column being produced by the kth tree given the observed data, as computed in equation (5). This posterior-weighting scheme is a common phylo-HMM training strategy. Intuitively, if we strongly believe that a column was generated by a particular tree, we would like the next update of that tree to have access to comparatively more information from that column than if that column-tree pair was unlikely. The higher a column is weighted for a particular tree, the more closely that tree will come to 'fitting' that column perfectly after the SEM step. In the extreme case where $P(x_m = k|D, \theta) = 1$ for certain columns and 0 for others, this is equivalent to performing Structural EM on only the columns for which $P(x_m = k|D, \theta) = 1$. As the EM training progresses, the hope is that the posterior distribution will become more and more certain of which trees go with which columns.

The above modified EM-algorithm is unusual in that the M-step is another EM. This is known as a nested EM, covered by [4]. This somewhat bends the rules of EM which state that there must be a reliable MLE for the model once the hidden data has been estimated. In this case, we note that EM typically increases drastically in likelihood in the first few iterations and then increases very slowly, so it is important to set the number of 'inner' iterations carefully. If the EM$_{inner}$ is allowed to go until it converges, then $P(D|\theta^{(n)})$ is guaranteed to increase monotonically with each iteration of EM$_{outer}$, but the speed of this convergence will be greatly compromised. If the EM$_{inner}$ is too restrained, irregular behaviour of $P(D|\theta^{(n)})$ may be observed, and convergence of EM$_{outer}$ is not guaranteed. Ideally, some middle ground would be reached in which the EM$_{inner}$ is allowed to run sufficiently so that EM$_{outer}$ increases in likelihood with every (or almost every) step [4]. In our programs, the EM$_{inner}$ is set to run 2 iterations of Structural EM which appears to be adequate. Figure **??** shows the likelihood progression of 6 trials from different start points on the same dataset. Note that each trial increases monotonically, except for a single 'dip' in likelihood midway through the 4th trial. In the points following this dip the likelihood is regained and convergence does not appear to be affected in the long run. The EM algorithm makes its greatest strides in the first few iterations, and then continues asymptotically towards the convergence value. Also note that it appears below as if

the value towards which the likelihood tends in each trial is independent of the start point. This is not in general the case, and running an effective analysis typically requires initializing the training scheme from several random start-points.

BEGIN EDIT

Algorithm: Posterior Decoding

Definitions: Let π be a path of length m through the state space of the HMM (discounting start and end states), emitting the first m columns of the alignment, with one state per column. Let M be the total number of columns in the alignment. We say that the state path is *partial* if $m \leq M$, and *complete* if $m = M$.

Let π_n denote the nth state in path π. The *score* of path π is defined as $S(\pi) = \sum_{n=1}^{m} P[\pi_n, n]$ where $P[i, m]$ is the posterior probability that column m was emitted by state i.

We say a state path π is *valid* if all its breakpoints are more than ϵ apart. That is, there exist no $m, n > 1$ with $n - m < \epsilon$ such that $\pi_{m-1} \neq \pi_m$ and $\pi_m \neq \pi_n$.

Let $\Pi(i, m, e)$ be the set of all valid partial state paths of length m, whose last e columns were emitted from state i. (That is, for $\pi \in \Pi(i, m, e)$ and $m - e < n \leq m$, we must have $\pi_n = i$.) We then define $U[i, m, e] = \max_{\pi \in \Pi(i,m,e)} S(\pi)$ to be the highest score of any such path, computed recursively as follows.

Input: A $k \times M$ matrix of posterior state-column probabilities, $P[i, m]$, and a minimum breakpoint separation ϵ.

Output: A complete valid state path π through the HMM such that the score $S(\pi)$ is maximal. Note that a state path uniquely determines a set of breakpoints $\{m : \pi_m \neq \pi_{m+1}\}$.

Recursion:

for $i = 1$ to k:

for $m = 1$ to M:

for $e = 1$ to $\min(m, \epsilon)$:

$$U[i, m, e] = P[i, m] + \max \begin{pmatrix} 0 & \text{if } m = 1 \\ U[i, m-1, e] & \text{if } m > 1 \\ U[i, m-1, e-1] & \text{if } m > 1 \text{ and } e > 1 \\ \max_{i'} U[i', m-1, \epsilon] & \text{if } m > \epsilon \text{ and } e = 1 \\ \max_{i'} U[i', m-1, m-1] & \text{if } 1 < m \leq \epsilon \text{ and } e = 1 \end{pmatrix}$$

Final score $U_{\text{final}} = \max_i U[i, M, 1]$

The score of the maximally-scoring path is U_{final}. The path having this score can be recovered by a straightforward traceback.

END EDIT

The terms in the max expression correspond to the possible incoming paths, and can be intuitively understood in the following way:

BEGIN EDIT

0 if $m = 1$: for the first column in the alignment, there is no incoming path. This term initializes the dynamic program.

$U[i, m-1, e]$ if $m > 1$: the path stays in the same state as the previous column. Since the incoming path was in state i for at least e steps, the current path must also have been in state i for at least e steps.

$U[i, m-1, e-1]$ if $m > 1$ and $e > 1$: the path stays in the same state as the previous column. Since the incoming path was in state i for at least $e - 1$ steps, the current path must have been in state i for at least e steps.

$\max_{i'} U[i', m-1, \epsilon]$ if $m > \epsilon$ and $e = 1$: the path changes state outside of the first ϵ columns of the alignment. To prevent breakpoints being closer than ϵ, this is only allowed to happen if the incoming path was in the same state for ϵ steps.

$\max_{i'} U[i', m-1, m-1]$ if $1 < m \leq \epsilon$ and $e = 1$: the path changes state within the first ϵ columns of the alignment. To prevent breakpoints being

closer than ϵ, this is only allowed to happen if the incoming path was in the same state for all $m - 1$ of the previous columns.

END EDIT

References

[1] Arenas M Posada D. *Recodon: Coalescent simulation of coding DNA sequences with recombination, migration and demography*. BMC Bioinformatics 458.8(2007).

[2] Durbin R, Eddy SR, Krogh A, Mitchison G. Biological Sequence Analysis. Cambridge, 1998.

[3] Hein J, Shierup H, Wiuf C. Gene Genealogies, Variation and Evolution. New York: Oxford, 2005.

[4] van Dyk D. *Nesting EM Algorithms for Computational Efficiency.* Statistica Sinica 10 (2000):203-225.

[5] Felsenstein J. *Evolutionary trees from DNA sequences: a maximum likelihood approach.* Journal of Molecular Evolution 17.6(1981):368-376.

[6] Friedman N, Ninio M, Pe'er I, Pupko T. *A Structural EM Algorithm for Phylogenetic Inference.* Journal of Computational Biology 9.2(2001):331-353.
